# Health Education Intervention as an Effective Means for Prevention of Respiratory Infections Among Hajj Pilgrims: A Review

**DOI:** 10.3389/fpubh.2020.00449

**Published:** 2020-09-03

**Authors:** Mohammed Dauda Goni, Habsah Hasan, Nadiah Wan-Arfah, Nyi Nyi Naing, Zakuan Zainy Deris, Wan Nor Arifin, Aisha Abubakar Baaba, Abdulwahab Aliyu, Babagana Mohammed Adam

**Affiliations:** ^1^Department of Microbiology and Parasitology, School of Medical Sciences, Universiti Sains Malaysia, Kubang Kerian, Malaysia; ^2^Faculty of Health Sciences, Universiti Sultan Zainal Abidin, Kuala Terengganu, Malaysia; ^3^Faculty of Medicine, Universiti Sultan Zainal Abidin, Kuala Terengganu, Malaysia; ^4^Unit of Biostatistics and Research Methodology, School of Medical Sciences, Universiti Sains Malaysia, Kubang Kerian, Malaysia; ^5^Centre for Language Studies and Generic Development, Universiti Malaysia Kelantan, Kota Bharu, Malaysia; ^6^Department of Pharmaceutical Microbiology and Biotechnology, Faculty of Pharmaceutical Sciences, Gombe State University, Gombe, Nigeria; ^7^African Institute of One Health Research and Diagnostics (AIOHRD), Abuja, Nigeria

**Keywords:** health education, respiratory infections, influenza, Hajj pilgrims, review

## Abstract

The prevalence of respiratory illness has continued to surge among Hajj pilgrims from different countries despite having some practices of preventive measures. Respiratory illnesses during Hajj could be due to many reasons and many factors that promote disease spread. These factors include overcrowding, cigarette smoking, and direct contact with infectious agents particularly viruses promote the spread of respiratory infections. However, due to the longer duration of the pilgrimage, there are high chances of pilgrims contracting various respiratory illnesses due to exposure to respiratory pathogens. Hajj pilgrims' knowledge, attitudes, and practices toward respiratory tract infections are used as the determinant of the effectiveness of the health education interventions. Knowledge and application of basic hygiene principles, use of face masks, following cough etiquettes, engaging in social distancing, and engaging in other measures are highly important. In this paper, we reviewed the various effective intervention strategies implemented to help prevent respiratory tract infections during Hajj.

## Introduction

The annual Hajj pilgrimage is one of the world's largest recurring mass-gathering events, with over three million pilgrims from almost 180 countries converging in Saudi Arabia (1). A mass gathering is defined by the World Health Organization (WHO) as an occasion, either organized or spontaneous, where the number of people attending is sufficient to strain the planning and response resources of the community, city, or nation hosting the event (2). The holy pilgrimage to Mecca, in Saudi Arabia, is among the five cardinal pillars of worship upon every financially and physically able Muslim individual. With the declaration of COVID-19 as a pandemic by the WHO (3), there are a total of 7,713,571 confirmed cases with a total mortality of 427,578 across the globe as at the time of writing this article on 14/06/2020 based on the data from Johns Hopkins University Coronavirus resource Centre, 2020 (4). Saudi Arabia, as the sole host of the Hajj pilgrimage, is also witnessing an increase in the trend of the COVID-19 infection (4). A higher number of the pilgrims involved in Hajj are elderly and also with underlying medical conditions, therefore at risk of the respiratory infections (5, 6).

Effective health education focused on prevention and control of respiratory tract infections during the Hajj pilgrimage depends on training, awareness, and compliance by Hajj pilgrims and healthcare workers involved (7, 8). Hajj pilgrims are at potential risk for several hazards and infections, and therefore, mitigation of these risks can be considered a most important international public health agenda by all stakeholders involved. Hajj pilgrims usually experience severe congestion, shared shelter, air pollution, and lack of proper hygiene; therefore, they easily get infected and transmit infections, most importantly acute respiratory infections (ARIs) (9). As a result, airborne infections are spread and transmitted easily to the various countries of the pilgrims upon their return (10). However, since the emergence of Middle East respiratory syndrome coronavirus (MERS-CoV), surveillance has been increased among pilgrims returning from Hajj in different countries (11).

Health education can be explained as a process by which individuals or groups learn to behave in a manner conducive to the promotion, maintenance, or restoration of health (12). An effective health education intervention has its own setbacks and challenges. The effectiveness of the intervention depends on its suitability to the target audience in their specific settings and taking into consideration their backgrounds (13). In 2007, an initiative called Health Education Ambassadors (HEA) was commissioned to provide health education for pilgrims upon their arrival during Hajj. The HEA are mandated with two cardinal objectives of providing effective health education for pilgrims in their mother tongue at their dormitories in the holy places and encourage medical students to actively take a health education role during the Hajj (14).

Respiratory tract infections are the most reported sicknesses during the Hajj pilgrimage which more often results in hospitalization. Pneumonia is the most common cause of severe sepsis and septic shock in patients admitted to intensive care units (ICUs) (15). Other conditions such as asthma, chronic obstructive pulmonary disease, and sinusitis could further produce complications by exacerbating the respiratory infections (16). The upper respiratory tract infections are associated with signs such as cough, sore throats, colds, tonsillitis, peritonsillar abscess, epiglottitis, laryngitis tracheitis, and hoarseness. The lower respiratory tracts infections are more often manifested as influenza, bronchiolitis, bronchitis, and pneumonia (17). The major viruses that serve as the etiological agents of such upper respiratory tract infection are influenza A and B viruses, adenovirus, coronavirus, rhinovirus, respiratory syncytial virus, enteroviruses, parainfluenza viruses, and Epstein–Barr virus (18). The most common bacterial causes of respiratory tract infection reported during Hajj pilgrimage from various studies are *Haemophilus influenzae, Klebsiella pneumoniae, Streptococcus pneumoniae, Staphylococcus aureus, Streptococcus pyogenes, Legionella pneumophila*, and *Mycoplasma pneumoniae* while pneumonia caused by tuberculosis is the common infection that leads to hospitalization (11, 19). The causative agent of severe community-acquired pneumonia during Hajj is compounded with inference regarding its prevention and control. Acute upper respiratory tract infections are the most common illnesses during the Hajj period (20).

The high prevalence of respiratory tract infections during Hajj are due to some factors such as cigarette smoking, direct contact with infected pilgrims, intermittent use of facemasks, and a failure to use alcohol-based hand disinfection. High-density crowds usually characterize this religious obligation and therefore pose a risk for the possible transmission and outbreaks of infectious agents. Over 90% of pilgrims are suffering from at least one respiratory symptom, which increases the risk of viral respiratory infections several fold (10). The cities hosting Hajj activities have been shown to have a higher prevalence of resistant tuberculosis than the annual risk of infection in other cities not involved with the Hajj ritual in the country. This may be the result of the number of pilgrims from countries where tuberculosis is endemic (21).

Since the emergence of confirmed cases of MERS-CoV in September 2012 in the Arabian Peninsula, pilgrims are faced with increased risk of contracting the infection and possibly importing it back to their various countries. Studies have indicated the occurrence of MERS-CoV in Europe, Asia, Africa, and North America either directly or indirectly as a result of direct or indirect links with the Middle East (22–24). Infection from MERS-CoV shows clinical signs and symptoms that have varied from asymptomatic to highly severe pneumonia with acute respiratory distress syndrome (ARDS), septic shock, and eventually death as a result of several organ failures (25). However, because of the current COVID-19 pandemic guidelines, the Kingdom of Saudi Arabia has taken some extraordinary steps to prevent the spread of COVID-19 (26).

## Methods

Published articles about “health education interventions” and “prevention of respiratory tract infections” during Hajj and Umrah were retrieved from ScienceDirect, Scopus, Cochrane databases, and PubMed using the words “Hajj and respiratory tract infections,” “influenza,” influenza-like illness,” and “health education.” A total of 150 articles written in English published between January 2000 and August 2018 were retrieved. These articles included randomized controlled trials (RCTs), quasi-experimental studies (non-RCTs), non-randomized trials, pre–post interventions with a control group, qualitative studies, cross-sectional studies, and prospective cohorts studies. The retrieved articles were then assessed for relevance. Published studies and reviews explored pre and post Hajj health education interventions for prevention of respiratory infections and literature covering various prevention guidelines such as use of facemasks, vaccination, cough etiquette, and social distancing during Hajj were included. Non-experimental studies such as reviews, letters, case reports, and systematic reviews were excluded from the review. Using the aforementioned criteria, a total of 19 articles were eventually selected for the present narrative review. Literature searches were independently done, and the title and abstracts were screened by two researchers for eligibility based on the inclusion criteria. Each of the selected articles was reviewed in full by two authors. The data were extracted from the included papers by two authors (AAB and AA). The extracted data were checked and verified by three authors (HH, NNN, and NWA) to ensure the accuracy of the data included.

## Results

### Health Education Interventions

Health education interventions have been proven to increase pilgrims' knowledge of respiratory illnesses (27). Information dissemination is a key factor in health education for mass gatherings, and it involves various modes, e.g., web-based, flip charts, lectures, discussions, symposia, posters, fliers, public addresses, and radio and television messages (14). The effectiveness of these various modes has both merits and limitation, which could determine the scope of their relative effect (28). Information may be passed to an intended audience but could be hindered by communication barriers and other factors such as cultural, psychological, and environmental issues. Therefore, the effectiveness of suitable health education interventions varies by the setting in which it is administered to a targeted group as shown in Table 1 (13). Depending on the availability of the delivery options for the module, different methods may be especially suitable for the delivery of health education interventions for different groups of people based on their age, level of education, and attained and cultural backgrounds (28).

**Table 1:** Health education intervention from various studies among Hajj pilgrims.

**References**	**Study design**	**Settings**	**Health Intervention**	**Outcome**
Abdin et al. (29)	Randomized controlled trial	Hajjis from Riyadh, Saudi Arabia, 2004 Hajj season	Health education for use of face mask and provision of free face mask	High compliance among the intervention group but no association between compliance and development of ARI
Choudhry et al. (30)	Prospective cohort study	Hajjis registered at primary healthcare centers of Riyadh, Saudi Arabia	Face mask	Face mask is significant against respiratory tract infection
Al-Zahrani et al. (31)	Cross-sectional study	International Arab pilgrims	Health education program	Health education is inadequate for pilgrims
Al-Asmary et al. (32)	Nested case–control study	250 personnel serving in a Hajj medical mission	Protective measures	Surgical mask use should be discontinued and regular use of alcohol-based hand scrubs
Ibrahim (33)	Cross-sectional study	Two randomly selected Mina hospitals in Makkah	Health education on vaccination, hand hygiene, face mask use, wearing wrist bandage, haircut, sleeping hours/day, and amount water/day	Health education before Hajj resulted in lowering the rate of risky behaviors
Gautret et al. (34)	Survey	Hajj pilgrims from Marseille, France, during the 2008 Hajj season	Low-cost physical measures, including use of face masks and hand hygiene	Adherence to preventive measures should be increased
Deris et al. (35)	Cross sectional study	Malaysian Hajj pilgrims 2007 season	Protective measures (vaccination and face mask)	Protective measures inadequate to give protection
Gautret et al. (36)	Observational study	405 French Hajj pilgrims 2009 season	Preventing measures against respiratory tract infections	Significance adherence to individual preventive measures
Balaban et al. (37)	Pre–post travel survey	US pilgrims from Michigan and Minnesota 2009 season	Protective practices recommended by CDC/WHO	40% of pilgrims reported respiratory illness
Emamian et al. (38)	Nested case–control design	Cohort consisting of 338 Iranian pilgrims	Preventive measures	Preventive measures have no effect on the incidence of respiratory tract infection
Elachola et al. (39)	Photo survey	All pilgrims passing through the Jeddah Airport Hajj terminal on arrival	Provision of face mask by NGOs and Saudi Arabia health ministry	Low compliance toward the use of face mask
Barasheed et al. (40)	Pilot study	164 Australian Hajj pilgrims during 2011 Hajj season	Effectiveness of face mask	No difference in occurrence of respiratory tract infection between intervention and control group based on laboratory results
Gautret et al. (41)	Pre–post travel questionnaire survey	French pilgrims, 2012–2014	Preventive measures	None of the preventive measures were effective in reducing cough prevalence
Wang et al. (42)	Cluster randomized controlled trial	1000 pilgrims from Saudi Arabia, Qatar, and Australia 2013 Hajj season. 2014/2015 Hajj pilgrims from Indonesia, Malaysia, Turkey, Morocco, India, Pakistan, and Bangladesh	3M™ Standard Tie-on surgical mask	Valuable evidence for the use of face mask was reported
Aelami et al. (43)	Prospective cross-sectional study	664 Iranian pilgrims during the 2012 Hajj season	Health education on personal hygiene including a hygienic package containing and alcohol-based handrub (gel or spray), surgical masks, soap, paper handkerchiefs, and user instructions	ILI was detected in 159 (52%) in the intervention group and 198 (55.3%) in the control group (p < 0.001). ILI was also observed less in pilgrims using a handrub in spray form (64; 41.4%) compared with those using a gel form (95; 61.2%)
Alqahtani et al. (27)	Cross-sectional survey	Australian Hajj pilgrims	Preventive measures through distribution of brochures	The study indicates that there are significant opportunities to improve awareness among Australian Hajj pilgrims about the importance of using preventive health measures
Hashim et al. (44)	Cross sectional study	Malaysian pilgrims during 2013 Hajj season	Preventive measures	Preventive measures inadequate, prevalence of respiratory illness remains high
Ramli et al. (45)	Quasi-experimental trial study	Malaysian men during 2010 Hajj season	Prevention practice through nasal rinsing five times a day	The study showed that nasal rinsing significantly reduced the symptoms of cough, rhinorrhea, and nasal blockage
Fahad et al. (46)	Intervention study	Hajj pilgrims from different countries at Makkah, Jeddah, and Medina	Health education programs	Health education strategy to pilgrims is effective in improving knowledge and practices and decreases the prevalence of health disorders among Hajj pilgrims

The use of smartphones across the globe is overwhelmingly associated with our daily routines and equally an essential part of present-day life, making it realistic as an effective means of delivering health education intervention and conducting prospective surveys among Hajj pilgrims through the use of smartphones (47). Therefore, data collection through smartphones may proffer a great and better avenue to carry out surveys and deliver education interventions among Hajj pilgrims than predominant paper-based survey methods (48). Several studies have clearly indicated the usefulness of delivering health education packages in different interventions and audiences with great response and degree of feedback (49).

Interventions are usually presented in the mass gathering care perspective due to the added advantage of ongoing professional support and monitoring, which could contribute to better results (50). Similarly, healthcare professionals and health educators are trained to deliver interventions to pilgrims through videos, posters, and fliers and through other means (14). Turkestani et al. recommended the use of health educational material for their possible protective measures. However, health education interventions were shown in a study to greatly improve participants' knowledge from 50% in pre-intervention to 84.7% post intervention in regard to dissemination of bloodborne diseases during Hajj (14). Therefore, health education delivered to pilgrims is effective in improving their short-term health knowledge.

Health education interventions about respiratory tract infection prevention based on WHO reports and guidelines for certain consideration for health promotion and prevention activities are optimum for achieving best preventive measures (51). The suggested guidelines are:

- identify, through risk assessment and historic surveillance, the most probable public health and communicable disease threats
- develop appropriate health promotion and prevention education messages and tools
- work with event organizers to promote and make available health information in event information packages for participants or visitors
- identify recommended, but not prescriptive, travel health recommendations—including for immunizations, safe practices (regarding sex, sharing water bottles, etc.), hand washing, cough etiquette
- offer practical advice on how to access medical assessment or services in the event of illness, and specific directions for doing so (e.g., call first before visiting hospitals)
- establish, and advertise the availability of, a toll-free health information line with interpretation capacity
- consider utilizing mobile public health intervention/response teams throughout the duration of the event (similar to the US National Disaster Medical System initiative for the Salt Lake City Winter Olympics in 2002)
- produce educational tools in multiple languages as required
- utilize multiple approaches for risk communication, including use of the Internet, and link online risk communication information to the main event website (51).

### Role of Health Education in Improving Knowledge, Attitudes, and Practices of Hajj Pilgrims for Prevention of RTI

Alqahtani et al. conducted an in-depth study with Australian Hajj pilgrims to learn the health knowledge, attitudes, beliefs, and practices prior to and after performing Hajj to determine the effectiveness of protective measures to curtail infectious diseases (27). The study showed that predeparture interventions presented by health educators, Hajj leaders, and medical personnel are crucial in helping promote compliance with preventive measures. This was very important in educating Australian Hajj pilgrims about the significance of using protective health measures. Similarly, a qualitative study among Australian pilgrims indicated a high level of awareness of the risk of acute respiratory infections (52). Another cross-sectional study conducted among Malaysian Hajj pilgrims revealed a good knowledge of RTIs about its prevention, although poor attitudes and practices were reported (53).

A study of KAP of health professionals regarding H1N1 showed that 42.9% of the respondents were unsure about the protection use of face masks would provide for them. In this study, 22.1% did not believe the protective role that washing hands with water and soap played, and 27.3% would settle for either option for protection. These results demonstrated that basic knowledge about protective measures against communicable diseases was significantly lacking among the respondents (54). A similar study conducted among Malaysian pilgrims was unable to document the benefit of influenza vaccination (55). However, Malaysian Hajj pilgrims showed a high prevalence of respiratory illness symptoms and the present preventive strategies seemed not enough to eradicate it (35). However, Al-Mohrej et al. suggested that knowledge of and attitudes toward respiratory illness such as MERS-CoV among the Hajj pilgrims are usually not up to the optimum level when compared to their practices of protective measures. Moreover, some false beliefs about treatment were common among the pilgrims. Studies of pilgrims from Turkey, France, and Australia indicated lack of knowledge and poor attitudes about respiratory tract infections, but French pilgrims showed great seriousness toward general protective procedures (14, 56–59). The KAP and awareness of Hajj pilgrims and healthcare personnel about basic hygiene principles and strategies should be emphasized through effective health education, and information dissemination strategies at the various preparatory stages prior to entry into Saudi Arabia have been shown to enhance pilgrims' knowledge, attitudes, and practices (56). An important vital factor for enhanced compliance lies in attitudinal and behavioral refinement (60). Pilgrims' knowledge, attitudes, and beliefs are regarded as having great influence on understanding of uptake of preventive measures and bridging the gap of delivering effective health information (52).

Protective behaviors based on the recommendation of community mitigation practices by WHO and the US Center for Disease Control and Prevention (CDC) are ideal for prevention of respiratory illnesses during Hajj and Umrah. Pilgrims from every country who have a history of serious medical conditions such as diabetes, hypertension, chronic lung disease, chronic renal disease, and immunodeficiency are at increased risk and are more susceptible to developing severe respiratory infections when exposed to the pathogens.

### Preventive Measures to Prevent Respiratory Tract Infections

#### Vaccination

The Saudi health authority has recommended annual seasonal influenza vaccination during each Hajj season to curtail the risk of spreading respiratory tract infections as far back as the 2005 Hajj season especially for those at high risk of influenza and other respiratory tract complications (61). The vaccination rates can be improved through proper and efficient implementation of strategies that include health education of caregivers and pilgrims to be vaccinated as a requisite for acquiring a Hajj visa. Currently, the health authorities in Saudi Arabia have recommendations for universal influenza vaccination for pilgrims coming for Hajj; however, it is pertinent that all pilgrims, including those who are less vulnerable, to receive the influenza vaccination (62). A study conducted with Malaysian pilgrims showed lower colonization rates among influenza-vaccinated pilgrims when compared with unvaccinated pilgrims though the rates were not statistically significant (44). The current recommendation from the Saudi Ministry of Health necessitates administration of meningococcal vaccine and seasonal influenza vaccination for all participating pilgrims across the globe prior to departure for Hajj. On the other hand, pneumococcal vaccination is not made compulsory for Hajj pilgrims but is highly recommended for those at risk for pneumococcal disease. Current pneumococcal vaccines used during Hajj have limited coverage of serotypes identified in Makkah (20). However, following the COVID-19 pandemic, up to date there is no vaccine available against the infection. In addition, all vaccines against pneumonia, such as pneumococcal vaccine and *Haemophilus influenza* type B (Hib) vaccine, do not provide protection against COVID-19 according to the WHO (63).

Among the 431 pilgrims from Australia in 2011, 278 (65%) were reported to have received influenza vaccine. However, with proper health intervention, uptake and coverage of vaccination substantially increased to 89% by the following year among the Australian pilgrims (40). A similar trend was observed among Iranian (86–98%), French 97%, local Saudi pilgrims (94%), and international pilgrims surveyed at airports (93.4%) (64–67). With these remarkable uptake and coverage figures, the prevalence of influenza-like illnesses showed a declining trend among Hajj pilgrims (68). Similarly, a study conducted among Malaysian Hajj and Umrah pilgrims showed a low uptake of influenza and pneumococcal vaccines were 28.6% (364/1,274) and 25.4% (324/1,274) respectively (6).

#### Hand Hygiene

Hand hygiene is considered and recognized across the globe as a basic, inexpensive, and non-pharmacological intervention recommended by various health organizations to help control and prevent the spread of influenza especially during pandemics (69). This is also commonly used as means of prevention of COVID-19 in addition to use of hand gloves. Hand hygiene is also one of the protective means to which the majority of pilgrims adhered (34). The use of a hand sanitizer during the pilgrimage is associated with a lower prevalence of *S. pneumonia* carriage (70). A survey of Australian and French pilgrims at the time of the 2014 and 2013 Hajj seasons reported that 94 and 50% of their pilgrims used various hand hygiene practices, which consisted of washing one's hands with soap, using water only to wash one's hands and the use of alcoholic hand disinfectant, respectively. Similarly, 67.2 and 57% of US and Turkish pilgrims showed a significant compliance with hand hygiene during the 2009 Hajj season, and this was positively associated with less risk of respiratory tract infection (37, 56). Similarly, the compliance with recommended hand hygiene among healthcare workers during 2009 influenza A pandemic was 97.5% (71). The cardinal motivations for these practices, according to the researchers, were based on the effectiveness of hand hygiene as a key component for prevention of respiratory diseases (27).

A peculiar feature is consistent hand washing during ablution at least five times a day prior to obligatory prayers, which is also considered as ritual purification (20). Generally, the use of alcoholic hand rubs for sustaining a good hand hygiene is pivotal in controlling respiratory viral infections but is not widely accepted among Muslim pilgrims due to the fact that alcohol is forbidden in Islam (60). Knowledge of Hajj pilgrims is poor in terms of the significance of hand hygiene in prevention of respiratory illnesses; however, compliance with hand washing is very good among the pilgrims based on the findings of the research (72).

#### Cough Etiquette

Covering of mouth and nose through proper use of a tissue when coughing or sneezing and its proper disposal in a receptacle bin after use are recommended by the CDC as preventive measures toward spreading respiratory tract infections. Most health authorities across the globe do not recommend covering the mouth/nose using bare hands when coughing because of possible dissemination of the pathogens (73). Cough etiquette as a means of protective behavior was reported as 46.2% among US Hajj pilgrims surveyed in 2009 while compliance among health workers was 89% during the same year, although there was no significant useful outcome about the prevalence of respiratory symptoms (37). The use of disposable tissues is preferred to cloth handkerchiefs for covering mouth and nose while coughing or sneezing because handkerchiefs can serve as a medium for breeding germs. Therefore, the handkerchief can spread the germs when you are sick and going around Hajj routines. A survey of pilgrims from France showed that knowledge of the use of handkerchief as a preventive measure against respiratory illnesses is very poor (1.1%); however, they had excellent intentions to use disposable handkerchiefs after being presented with an educational intervention and health promotion was given to them (34). Another study conducted among Malaysian men during the 2010 Hajj season demonstrated that nasal rinsing is significant in reducing the symptoms of cough, rhinorrhea, and nasal blockage (45). The WHO also strongly recommends cough etiquette to reduce the general risk of transmission of acute respiratory infections, including by the virus causing COVID-19 (74).

#### Contact Avoidance and Social Distancing

Crowdedness is a common occurrence peculiar with all the rituals involved during Hajj pilgrimages and poses a major risk for the spread of respiratory illness. Therefore, it becomes unrealistic to properly implement contact avoidance as a means of preventing respiratory illnesses (8). According to CDC, limiting contact with others is the best way to reduce the spread of coronavirus disease 2019 (COVID-19). Similarly, Saudi Arabia health authorities have introduced social distancing measures for worshippers during prayers in the Grand Mosque and other Holy sites due to the COVID-19 pandemic (75). Although no Hajj pilgrimage is yet to be conducted while practicing social distancing or crowd avoidance.

Pilgrims who participated in the 2009 Hajj had poor knowledge of avoiding crowds or public gathering as means of preventing spread of HIN1 (18%) (76). As reported in a study of US pilgrims, those who engaged in practicing social distancing (34.4%) and contact avoidance (24.2%) reported fewer cases of respiratory illnesses and were associated with shorter respiratory illnesses duration (37). Hajj pilgrims surveyed in different studies reported that 82% of Arab pilgrims, 48% of Turkish pilgrims, 86% of French pilgrims, and 73% of Australian pilgrims all perceived that avoiding contact with sick people was a key element that would reduce transmission of infections (56). However, a nested case–control study conducted with Iranian Hajj pilgrims in 2010 revealed no association between direct contact with sick pilgrims with average daily presence in the holy areas and respiratory tract infections (38).

#### Face Mask

Proper usage of face masks can reduce the risk of contracting respiratory pathogens and also is a potentially effective preventive strategy against the transmission and dissemination of “Hajj cough” (41). However, its effectiveness of use by Hajj pilgrims depends on the type, design, quality, and proper usage of the face mask. Several studies of pilgrims from different countries have shown that the effect of face masks in helping prevent respiratory tract infections during Hajj is not achievable and remains unknown due to some improper usage and compliance (35, 77). Uptake of face mask use among pilgrims showed an increase up to 64% in 2014 (33). A high compliance was shown in a study conducted on Hajj pilgrims but no association between the compliance and development of acute respiratory symptoms among the participants (29). Furthermore, the compliance of pilgrims for the use of face masks in the course of Hajj became high (91.7%) as a result of health promotion interventions about their use (78). On the other hand, while face mask use compliance is generally poor among Hajj pilgrims from various countries; Malaysian, French, and Iranian pilgrims recorded over a 60% compliance rate (10). The use of a facemask by men, but not use of a face cover by women, was also reported to be a significant protective factor against respiratory infections (30). Laboratory findings from a study for the effectiveness of face mask use among Australian pilgrims showed no difference between the intervention and control group (79).

The CDC does not advocate face mask use during mass gatherings as a means of preventing respiratory tract infections except for any participant who becomes sick at your event (80). However, in April 2020, the CDC recommended the use of cloth face masks to curtail community-based transmission (81). In the heat of the COVID-19 pandemic, the use of the face mask varies across countries and therefore remains debatable (82). The WHO, in its interim guidance released in April 2020, did not recommend the mass use of face masks for healthy individuals in the community (mass masking) as a way to prevent infection with COVID-19 (83).

## Discussion

“Health education can be explained as a process by which individuals or groups learn to behave in a manner conducive to the promotion, maintenance, or restoration of health” (28). Based on our extensive literature review, various interventions can be used for health education in promoting awareness for the pilgrims such as lectures, discussions, symposia, posters, public addresses, and radio and television messages depending on the age, sex, educational level, background of the individual, and nature of the individual's employment (13). In non-governmental organizations and agencies under the government, health education interventions can help societies determine its needs, work on developing its problem-solving strengths, and set in motion various means to develop, disseminate, implement, evaluate, and comply with the strategies to reform the health status of the society (84). Findings from a study of a small web-based intervention to reduce transmission of influenza showed trends in behavior change, but no effect on hand hygiene (85). Non-pharmaceutical interventions are often considered because they are relatively less expensive and non-invasive methods to address mortality and morbidity from influenza-like illnesses (86).

Health education packages and awareness kits are designed in such a way that cultural, ethnic, and language diversity are prioritized for the prevention of infectious diseases during Hajj. The Saudi Ministry of Health partners with relevant authorities in various countries that have annual pilgrims to map out and produce appropriate interventions for education and awareness including vaccination guidelines and the precautions to be taken prior to departure to Saudi Arabia and during the Hajj. Various health educational materials (guides, pocket cards, stickers, leaflets, fliers, and posters) have been developed in English, Arabic, Urdu, and several other languages (87). However, training and educating healthcare workers and professionals who are always the first to encounter cases during the pilgrimage have been paramount in terms of the effectiveness of prevention of respiratory tract infections. The training of the healthcare workers will ensure prevention and spread of diseases through early detection and contact tracing (88).

Recently, few published studies have shown the importance of educational interventions in the improving Hajj pilgrims' knowledge, attitudes, and beliefs about respiratory infections. Alqahtani et al. (27) found that substantial misunderstandings about protective measures and the dangers of respiratory infections persist among Hajj pilgrims from Australia. Similarly, Gautret et al. (66) showed that French Hajj pilgrims were familiar with the preventive measures for respiratory infections such as use of hand disinfectant (77.4%), use of disposable handkerchiefs (89.8%), and use of face masks (79.6%). In addition, 97.4% of the French pilgrims were vaccinated against seasonal flu, 5.8% against H1N1, and 31.4% against pneumococcus. However, no work has been published regarding the barriers to and facilitators of the uptake of preventive measures. However, a study conducted among international Arab pilgrims showed that health education is inadequate for improving their knowledge of health hazards (31).

The Saudi Health Authority issues current updated Hajj travel guides and health regulations via a network of international public health agencies such as the WHO, the CDC, and Hajj travel companies (89). Ministries of health in countries of pilgrims' origin are mandated to provide information, promote awareness, and offer campaigns to pilgrims about communicable diseases that include symptoms, means of transmission, complications, and prevention guides (90). These authorities have rolled out several series of programs to educate and enlighten the pilgrims in such areas as infection control practices (e.g., use of face masks) to control the occurrence of serious Hajj-related illnesses (89). It is important and very critical for every participating country to properly and prepare its pilgrims as a preamble prior to departing for Hajj as collectivism is required from all participating countries to address the multiple challenges. Healthcare professionals, private Hajj operators, statutory bodies, and community collaborative efforts are highly vital in ensuring well-coordinated Hajj rites. The effectiveness of health promotion and the regular use of standardized infection prevention protocols such as hand hygiene, and more advanced measures have been demonstrated in hospital-associated MERS-CoV outbreaks in Saudi Arabia and in Korea (91).

In a study of Iranian pilgrims, 664 pilgrims were randomly assigned to an intervention group (306) and a control group (358). The intervention group was provided an education intervention about personal hygiene while the control group did not receive any educational intervention. The results showed that influenza-like illnesses (ILI) were observed less in pilgrims using a hand rub in spray form (64; 41.4%) compared with those using a gel form (95; 61.2%). Hygienic education intervention, together with the provision of a health package including face masks, paper handkerchiefs, soap, and a hand rub, was provided, and these can help prevent influenza like illnesses among pilgrims (43).

A health education intervention was conducted in a study through a pre- and post-intervention study design of six buses selected at random from a total of about 300 buses with a sample size of 300 participants at the King Abdul Aziz International Airport. A validated questionnaire was administered to the participants to determine their knowledge for health behavior during Hajj and shown to be effective in increasing short-term health knowledge (14).

### Future Plan for Hajj and Umrah Following the COVID-19 Pandemic

Since the emergence of the COVID-19 pandemic, health authorities in Saudi Arabia and other participating countries have started planning for the well-being of pilgrims to help ensure public health. One of the measures for preventing the spread of the COVID-19 infection is the prohibition of mass gatherings. However, Hajj and Umrah pilgrimage is usually associated with a massive number of participants. Therefore, plans should be toward limiting the number of pilgrims to curtail the spread COVID-19 during the pandemic. Countries participating in the Hajj, as well as the host country, should focus on educating the pilgrims and the community about the critical component of the interventions that were established from previous studies that are significant in reducing the incidence of the respiratory tract infections. The initial decision is the immediate cancellation of the Umrah pilgrimage and suspension of the daily prayers at the Grand Mosque of Mecca and Medina. If restriction of travel into the Kingdom of Saudi Arabia becomes successful in the prevention of the spread of the virus, it is predicted that authorities may have to cancel the entry of pilgrims for Hajj 2020 (92). Similarly, various participating countries have taken steps amid the pandemic to prevent viral spread. Indonesia, the largest Muslim population nation with more than 220,000 pilgrims participating in the 2020 Hajj pilgrimage have canceled plans to attend the 2020 Hajj pilgrimage due to the COVID-19 pandemic (93).

## Conclusion

The majority of the published studies we reviewed for this paper showed that there is still a need to educate and enhance the awareness of the significance of using preventive health guidelines among Hajj pilgrims. Due to the overwhelming task involved in Hajj preparations, simplified and more robust health education interventions will, in no small measure, contribute to the public health sector and ease the challenges faced by pilgrims. Health education interventions based on scientifically proven measures such as vaccination for influenza-like illnesses can be effective but may not be achievable at the initial stages in case of pandemic influenza outbreak. Delivery of the health education interventions via smartphone applications is by far easier in reaching wider coverage and producing quick feedback. Even though there are persistent knowledge gaps in bridging the relative effectiveness between intervention and control groups, several researchers have suggested that campaigns and awareness for further enhancement of frequency of hand hygiene, coupled with use of facemasks in situations with a high risk of exposure as seen during the Hajj pilgrimage, are likely to contribute to prevention of respiratory tract infections. To ensure optimum prevention, all preventive measures must be practiced together to ensure they complement one another as a cluster of care to help prevent respiratory illness effectively. These health interventions are programs that should be done before the pilgrimage, during the pilgrimage, and even after returning back from the pilgrimage.

## Author Contributions

MG and ZD conducted the survey and drafted the initial manuscript. NN, HH, NW-A, and WA designed and supervised the study. AB and AA helped in the manuscript revision. All authors read and approved the final manuscript. BA contributed in the draft of the initial manuscript.

## Conflict of Interest

The authors declare that the research was conducted in the absence of any commercial or financial relationships that could be construed as a potential conflict of interest.
