# Supplementary material for: Health Education Intervention as an Effective Means for Prevention of Respiratory Infections Among Hajj Pilgrims: A Review
Source: Front Public Health. 2020 Sep 3;8:449. doi: 10.3389/fpubh.2020.00449 (PMC7494962; doi:10.3389/fpubh.2020.00449)
Supplement: Supplementary file 1 [file Data_Sheet_1.docx]

**Table 1 Health educational interventions among Hajj pilgrims from various studies**

| Author | Study design | Settings | Health Intervention | Outcome |
| --- | --- | --- | --- | --- |
| Abdin *et al.,* 2005 | Randomized controlled trial | Hajjis from Riyadh, Saudi Arabia 2004 hajj season | Health education for use of face mask and provision of free face mask | High compliance among the intervention group but no association between compliance and development of ARI |
| Balaban *et al.,* 2012 | Pre-post travel survey | US pilgrims from Michigan and Minnesota 2009 season | Protective practices recommended by CDC/WHO | 40% of pilgrims reported respiratory illness |
| Gautret *et al*., 2015 | Pre-post travel questionnaire survey | French pilgrims, 2012–2014 | Preventive measures | None of the preventive measures were effective in reducing cough prevalence |
| Wang *et al.,* 2015 | Cluster randomized controlled trial | 1000 pilgrims from Saudi Arabia, Qatar and Australia 2013 Hajj season. 2014/2015 hajj pilgrims from Indonesia, Malaysia, Turkey, Morocco, India, Pakistan and  Bangladesh | 3M^TM^ Standard Tie-on surgical mask | Valuable evidence for the use of face mask was reported |
| Ramli *et al*., 2018 | Quasi experimental trial study | Malaysian men during 2010 Hajj season | Prevention practice through Nasal rinsing five times a day | The study showed that nasal rinsing significantly reduced the symptoms of cough, rhinorrhoea, and nasal blockage |
| Alamri et al., 2018 | Intervention study | Hajj pilgrims from different countries at Makkah, Jeddah and Medina | Health education programs | Health educational strategy to pilgrims is effective in improving knowledge and practice and decreases the prevalence of health disorders among Hajj pilgrims. |
